# Analysis of the effect of NEKs on the prognosis of patients with non-small-cell lung carcinoma based on bioinformatics

**DOI:** 10.1038/s41598-022-05728-4

**Published:** 2022-02-01

**Authors:** Mengxia Yang, Yikun Guo, Xiaofei Guo, Yun Mao, Shijie Zhu, Ningjun Wang, Dianrong Lu

**Affiliations:** 1 Graduate School, Beijing University of Chinese Medicine, Beijing, 100029 People’s Republic of China; 2 Department of Oncology, Wangjing Hospital, China Academy of Chinese Medical Sciences, Beijing, 100102 People’s Republic of China; 3 Department of Oncology, The Second Hospital of Hunan University of Chinese Medicine, Changsha, 410005 People’s Republic of China

**Keywords:** Cancer, Lung cancer, Tumour biomarkers

## Abstract

NEKs are proteins that are involved in various cell processes and play important roles in the formation and development of cancer. However, few studies have examined the role of NEKs in the development of non-small-cell lung carcinoma (NSCLC). To address this problem, the Oncomine, UALCAN, and the Human Protein Atlas databases were used to analyze differential NEK expression and its clinicopathological parameters, while the Kaplan–Meier, cBioPortal, GEPIA, and DAVID databases were used to analyze survival, gene mutations, similar genes, and biological enrichments. The rate of NEK family gene mutation was high (> 50%) in patients with NSCLC, in which NEK2/4/6/8/ was overexpressed and significantly correlated with tumor stage and nodal metastasis status. In addition, the high expression of NEK2/3mRNA was significantly associated with poor prognosis in patients with NSCLC, while high expression of NEK1/4/6/7/8/9/10/11mRNA was associated with good prognosis. In summary, these results suggest that NEK2/4/6/8 may be a potential prognostic biomarker for the survival of patients with NSCLC.

## Introduction

Globally, lung cancer remains a major problem affecting human health, and its morbidity and mortality are continuously increasing^1,2^. According to GLOBOCAN statistics, over 2.09 million new cases of lung cancer were reported in 2018, accounting for 11.6% of the total cancer cases, and 1.76 million lung cancer-related deaths occurred, accounting for 18.4% of the total cancer deaths^3^. Non-small cell lung cancer (NSCLC) accounts for 85% of all lung cancer cases^4^, of which 25%–30% are squamous cell carcinomas^5^ and 40%–45% are adenocarcinomas^6^. Overall, the 5-year survival rate of lung cancer is relatively low (approximately 19%)^2^. The main reason for this is that most patients are diagnosed at later stages and receive insufficient treatment^7^. Some previous studies have shown that some biomarkers can contribute to the rapid clinical diagnosis of NSCLC^8^. Therefore, there is an urgent need to develop a prognostic marker for NSCLC with potential clinical value in order to provide new ideas and methods for its early diagnosis and treatment.

NEKs, also known as NIMA-related kinases, consist of 11 different family members that have N-terminal catalytic domains and encode different serine/threonine kinases^9^. Except for NEK11, they can be divided into four groups according to their specificity for serine/threonine phosphate receptors and their preference for acid/basic residues other than the 3-hydrophobic residue, namely, NEK1/3/4, NEK6/7/9, NEK5/8, and NEK2/10^10^. NEKs are proteins that are involved in various cell processes, such as cell cycle, mitosis, cilia formation, and DNA damage response; participate in cell differentiation and maintenance of cell homeostasis; and play important roles in the formation and development of cancer^11^. For example, NEK2/5 is associated with cell death resistance^12,13^, of which NEK2 is significantly associated with lung cancer^14^ and NEK5 expression is associated with breast cancer^15^. However, these studies mainly focused on a gene belonging to the NEK family, and the mechanism of all members of the family in the development of NSCLC has not been reported.

In this study, we aimed to comprehensively analyze the expression, mutation, and clinical prognosis of NEK family members in patients with NSCLC in order to explore their potential clinical values.

## Materials and methods

Analysis of differential expression and pathological parameters. In order to analyze the expression of NEKs in NSCLC, we obtained the data from the Oncomine and UALCAN databases and visualized the expression profiles. The Oncomine gene expression microarray dataset (www.oncomine.org) is a genome-wide expression dataset and a Web-based data-mining platform^16^ that can be used to detect the mRNA expression of NEK family members in 20 types of tumors. Using “NEKs” as the key word, the analysis type was “cancer and normal tissue analysis,” the setting parameter was a P < 0.01, the fold change was > 1.5, the gene rank was 10%, and the data type was mRNA. In addition, we analyzed the expression of NEK mRNA and its relationship with clinicopathological parameters such as tumor stage and nodal metastasis status in patients with LUAD and LUSC using the UALCAN database (http://ualcan.path.uab.edu)^17^.

Immunohistochemical analysis. The Human Protein Atlas (HPA, https://www.proteinatlas.org/) is a database for the systematic study of human proteomes based on immunohistochemical analysis^18^. In this study, we obtained the expression patterns of NEKs proteins in human normal lung tissues and lung cancer tissues from this database and evaluated them as high, medium, low, and not detected according to the staining intensity.

Overall survival analysis. The Kaplan–Meier Plotter database (https://kmplot.com/analysis/) collects the survival data from the GEO and TCGA databases. It can study more than 54,000 genes and 21 types of cancers and analyze the prognosis of malignant tumors online. In this study, we selected "auto select best cutoff" as the cutoff value and used this database to analyze the prognostic value of NEKs in patients with NSCLC.

Mutation analysis. The cBioPortal database (https://www.cbioportal.org/)^19^ can be used for interactive exploration of multiple cancer genomics datasets. Its data come from the TCGA, Oncomine, and other data platforms and can be used to study somatic mutations, DNA copy number mutations, DNA methylation, and other gene types. In this study, we used this database to analyze the genetic mutations of NEKs; the selected data set was “lung adenocarcinoma/lung squamous cell carcinoma (LUAD/LUSC) (TCGA, Firehose Legancy),” the name of the sample was “sample with mRNA data (RNA Seq V2,” z-score threshold =  ± 1.8)”, and the gene name “NEKs” was entered to conduct the search.

Bioinformatic analysis of similar genes. GEPIA 2.0 (http://gepia.cancer-pku.cn/) was used to determine 50 adjacent genes that were significantly related to NEK mutations in NSCLC patients^20^, while STRING11.0 database (https://string-db.org/) was used to analyze these similar genes, whose screening condition was with an interaction score of 0.9^21^. The MCODE plug-in in the Cytoscape software (version 3.8.1; www.cytoscape.org) was used to filter out important modules using the following filter conditions: “Degree cutoff = 2,” “Node score cutoff = 0.2,” “K-core = 2,” and “Max depth = 100.” In addition, GO, KEGG, and RECTOME enrichment analyses of NEKs and similar genes were performed using the DAVID database (https://david.ncifcrf.gov/summary.jsp)^22^.

## Results

**Figure 1:**
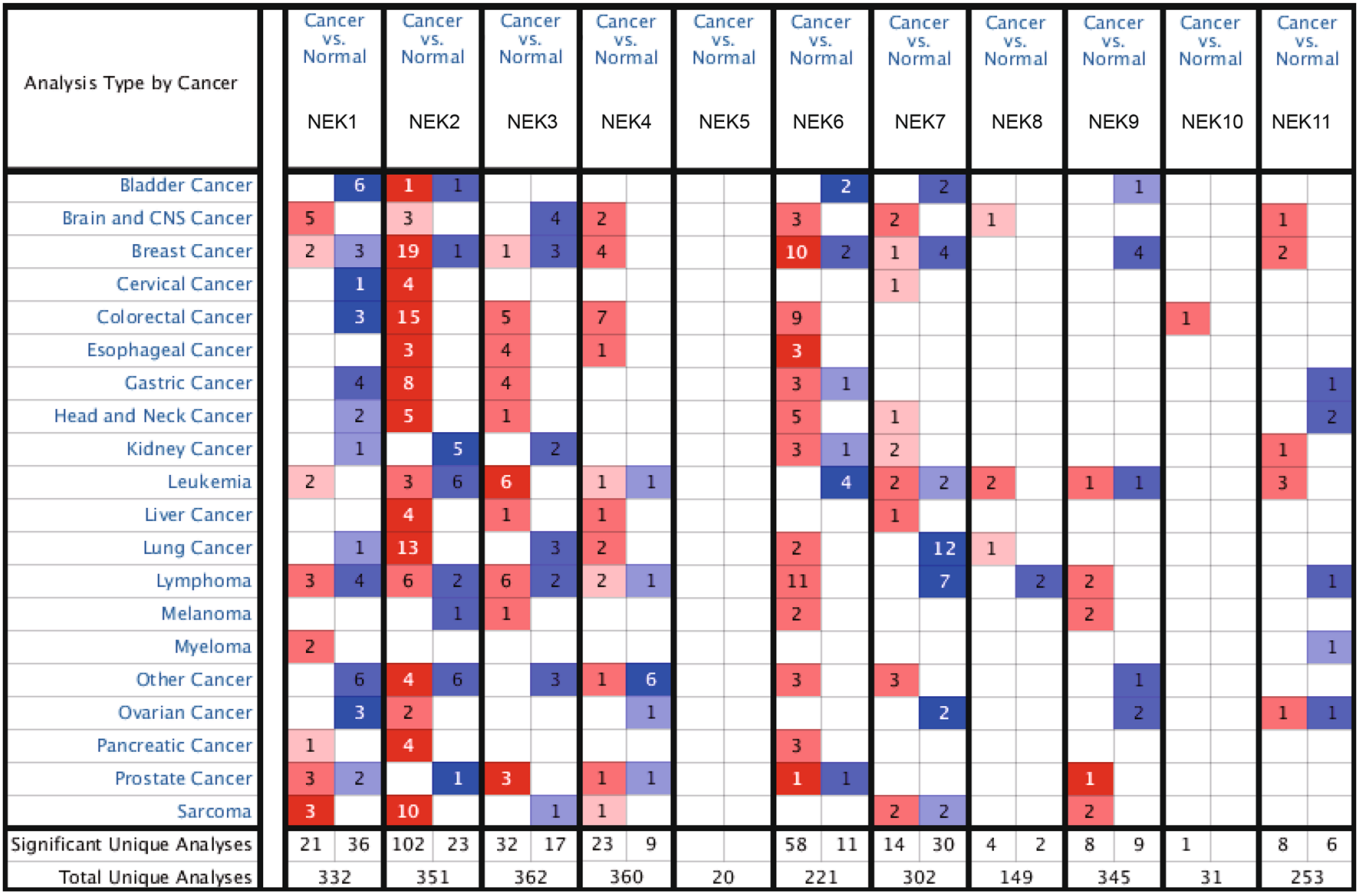
Transcriptional expression of NEKs in 20 different types of cancer diseases.

**Figure 2:**
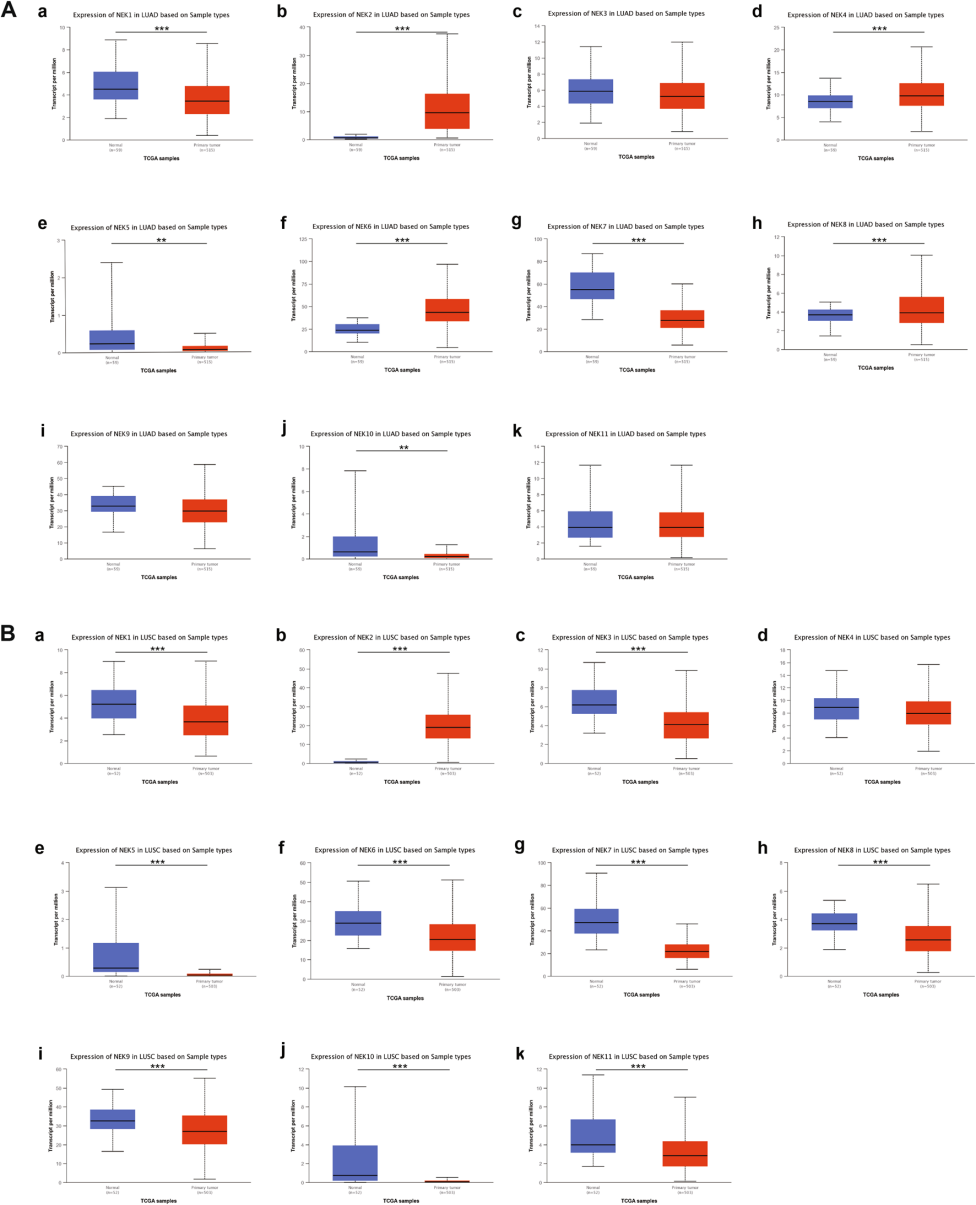
mRNA expression of distinct NEKs family members in NSCLC tissues and and normal lung tissues. *P < 0.05, **P < 0.01, ***P < 0.001. **A** From a to k represent the mRNA expression of NEK1 to NEK11 in LUAD tissues and normal lung tissues; **B** From a to k represent the mRNA expression of NEK1 to NEK11 in LUSC tissues and normal lung tissues.

Overexpression of NEK mRNA in NSCLC. We used the Oncomine database to measure the mRNA expression levels of NEKs in 20 cancer tissues. Results showed that there was overexpression of NEK2/4/6/8, low expression of NEK1/3/7, and no expression of NEK5/9/10/11 in lung cancer (Fig. 1). In addition, multiple data sets showed a significant increase in the expression of NEK2/4/6/8mRNA in lung cancer tissues (Table 1). We used the UALCAN database to detect the expression of NEK mRNA and found that there was a significant overexpression of mRNA in NEK2/4/6/8 in patients with LUAD and NEK2 mRNA in patients with LUSC (*P* < 0.01) (Fig. 2).

**Table 1:** Significant changes of NEKs expression in transcription level between NSCLC and normal lung tissues.

	Gene	Fold Change	*P* value	t-test	References
LUAD	NEK2	5.492	1.07E−06	9.327	Garber Lung^23^
		5.771	6.84E−17	11.903	Hou Lung^24^
		8.410	2.83E−09	6.940	Su Lung^25^
		2.861	2.64E−16	10.743	Landi Lung^26^
		3.038	5.67E−14	10.944	Okayama Lung^27^
		2.188	6.16E−13	8.980	Selamat Lung^28^
	NEK4	2.145	0.005	2.833	Bhattacharjee Lung^29^
	NEK6	2.138	1.96E−14	12.855	Okayama Lung^27^
		1.891	7.03E−15	9.080	Selamat Lung^28^
	NEK8	1.777	1.43E−10	7.034	Selamat Lung^28^
LUSC	NEK2	9.356	6.95E−32	27.482	Hou Lung^24^
		4.244	6.21E−06	7.801	Garber Lung^23^
		2.508	0.002	5.664	Wachi Lung^30^

**Figure 3:**
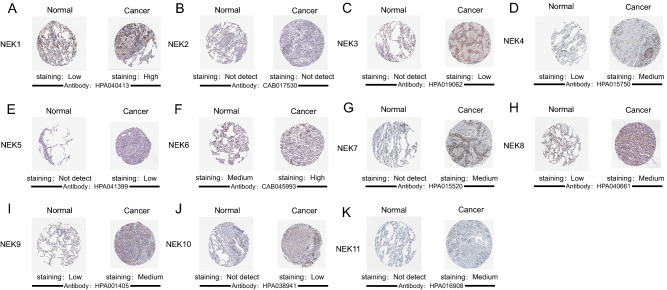
Representative immunohistochemistry images of distinct NEKs family members in NSCLC and normal lung tissues. From **A** to **K** represent the representative immunohistochemistry images of NEK1 to NEK11 in NSCLC and normal lung tissues.

Overexpression of NEK proteins in NSCLC. We used the HPA database to explore the protein expression levels of NEKs in NSCLC. We found that NEK2 protein was not expressed in normal tissues and NSCLC, and the protein expression levels of other NEKs in NSCLC showed higher expression levels than those in normal tissues. The NEK3/5/7/10/11 proteins showed no expression in normal tissues, but low and moderate expression in NSCLC. The NEK1/4/6/8/9 proteins showed low and medium expression in normal tissues, but high and medium expression in NSCLC (Fig. 3).

**Figure 4:**
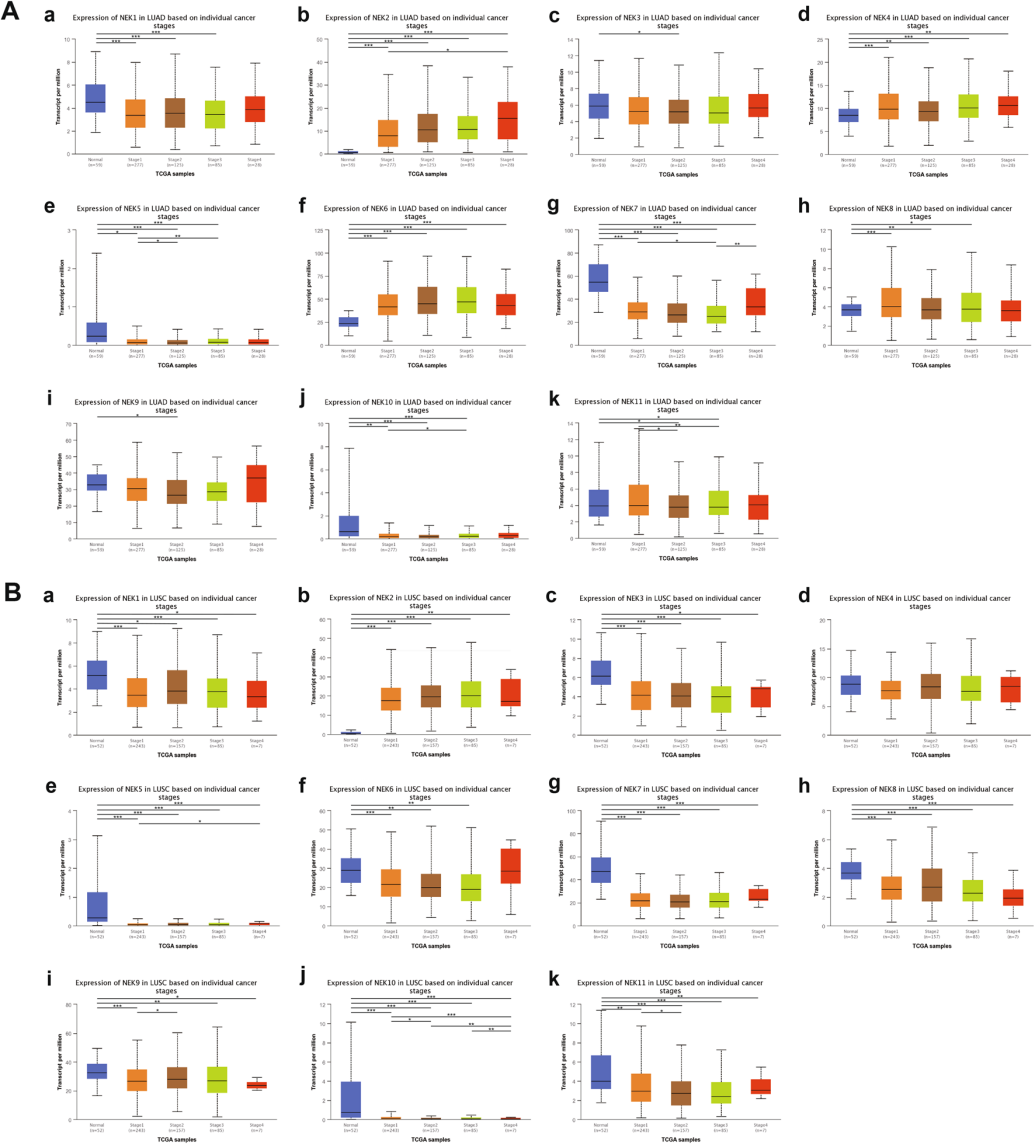
Relationship between mRNA expression of distinct NEKs family members and individual cancer stages of NSCLC patients. **A** From a to k represent the relationship between mRNA expression of NEK1 to NEK11 and individual cancer stages of LUAD patients; **B** From a to k represent the relationship between mRNA expression of NEK1 to NEK11 and individual cancer stages of LUSC patients.

**Figure 5:**
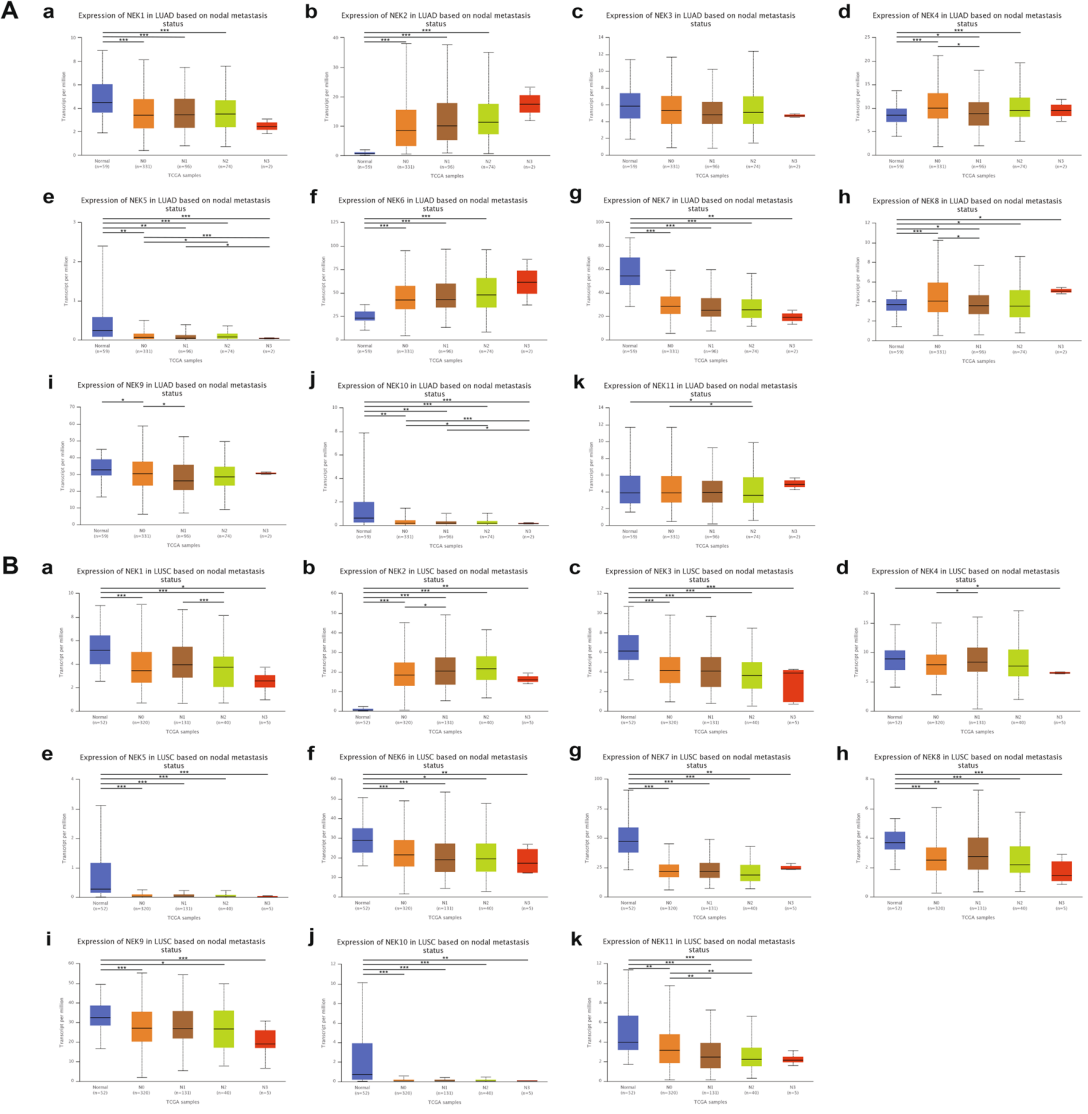
Relationship between mRNA expression of distinct NEKs family members and individual nodal metastasis status of NSCLC patients. **A** From a to k represent the relationship between mRNA expression of NEK1 to NEK11 and individual nodal metastasis status of LUAD patients; **B** From a to k represent the relationship between mRNA expression of NEK1 to NEK11 and individual nodal metastasis status of LUSC patients.

Analysis of the relationship between NEKs and clinicopathological parameters. We used the UALCAN database to analyze the relationships between NEK mRNA expression levels and the bedside pathological parameters of NSCLC patients, including tumor stage and nodal metastasis status. With regard to tumor stage, the expression of NEK mRNA was significantly correlated with the tumor stage of patients. Results showed that in LUAD patients, the highest expression levels of NEK1/2/3/4/7/9/10/11 mRNA were observed in those with stage 4, while the highest expression levels of NEK5/6/8 mRNA were observed in those with stages 3, 2, and 1, respectively (Fig. 4A). Meanwhile, patients with LUSC had the highest second-phase mRNA expression levels of NEK1/2/8/9/10 and the highest fourth-phase mRNA expression levels of NEK3/4/5/6/7/11 (Fig. 4B). In terms of nodal metastasis status, NEK2/4/6/8 mRNA was overexpressed in LUAD patients, and their highest expression levels were in N3 (Fig. 5A). In patients with LUSC, only the mRNA of NEK2 was overexpressed (Fig. 5B).

**Figure 6:**
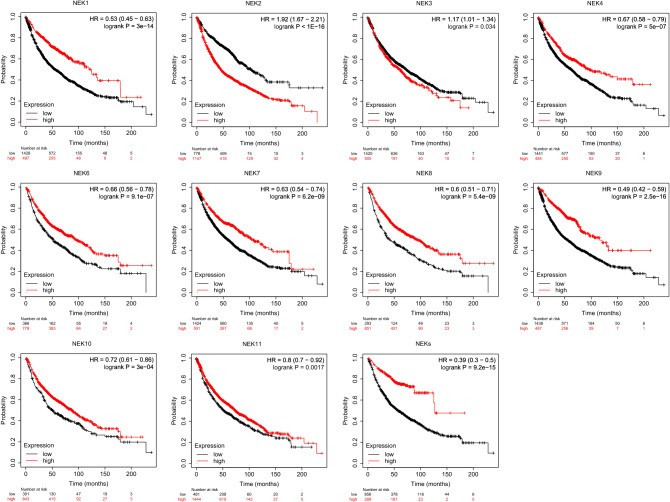
Prognostic value of mRNA expression of distinct NEKs family members in NSCLC patients.

Prognostic values of NEK mRNA expression in NSCLC patients. We used the Kaplan–Meier plotter database to analyze the effect of NEK mRNA expression on the prognosis of patients with NSCLC. Results showed that the expression of NEK mRNA was significantly correlated with the prognosis of patients with NSCLC, but NEK5 could not find the related prognostic information. Among them, the high mRNA expression of NEK family members is related to the long-term prognosis of NSCLC patients (hazard ratio [HR] = 0.39, 95% confidence interval [CI]: 0.30–0.50, P = 9.2E–15). Further analysis of the relationship between the expression of mRNA in different NEKs and the prognosis of NSCLC patients showed that the mRNA expression level of high NEK2 (HR = 1.92, 95% CI 1.67–2.21, P = 1E–16) and NEK3 (HR = 1.17, 95% CI 1.01–1.34) was associated with poor prognosis. The mRNA expression levels of high NEK1 (HR = 0.53, 95% CI 0.45–0.63), NEK4 (HR = 0.67, 95% CI 0.58–0.79), NEK6 (HR = 0.66, 95% CI 0.56–0.78), NEK7 (HR = 0.63, 95% CI 0.54–0.74), NEK8 (HR = 0.6, 95% CI 0.51–0.71), NEK9 (HR = 0.49, 95% CI 0.42–0.59, P = 222.5E–16), NEK10 (HR = 0.72, 95% CI 0.61–0.86, P = 3E–04), and NEK11 (HR = 0.8,95% CI 0.70–0.92) were significantly associated with good prognosis (Fig. 6).

**Figure 7:**
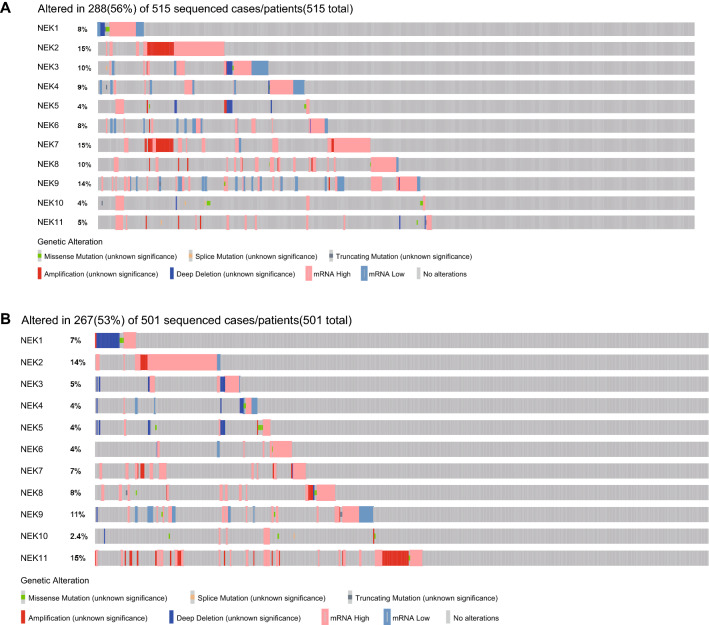
Genetic mutations in NEKs. **A** Mutations of NEK1 to NEK11 in LUAD patients; **B** Mutations of NEK1 to NEK11 in LUSC patients.

NEKs gene mutations. We used the cBioPortal database to analyze the gene mutations of NEKs. Among the 515 LUAD patients, 288 patients had gene mutations, and the mutation rate was 56%; in 501 LUSC patients, 267 had gene mutations, and the mutation rate was 53%. Among LUAD patients, the mutation rates of NEK/2/7/9 were the highest, at 15%, 15%, and 14%, respectively. Among the patients with LUSC sequencing, the NEK2/9/11 gene mutation rates were the highest, at 14%, 11%, and 15%, respectively (Fig. 7).

**Figure 8:**
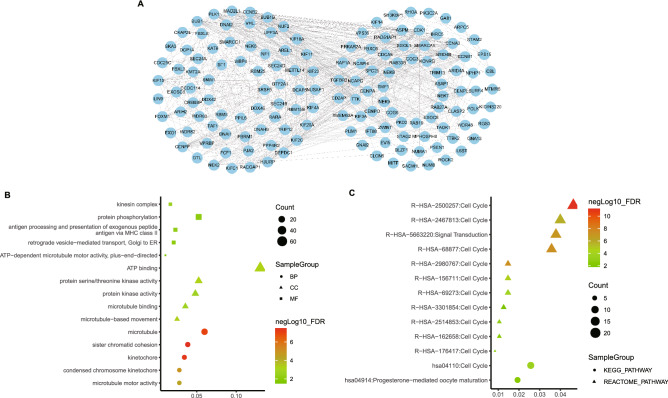
Predicted functions and pathways of the mutations in NEKs and their 50 frequently altered neighbor genes in NSCLC patients. Network of NEKs mutations and their 50 frequently altered neighbor genes was constructed (**A**). GO functional enrichment analysis predicted three main functions of NEKs mutations and their 50 frequently altered neighbor genes, including biological process, cellular components and molecular functions (**B**). KEGG pathway analysis on NEKs and their 50 most frequently altered neighbor genes was shown at figure (**C**).

Functional enrichment analysis of NEKs and similar genes. We used GEPIA 2.0 to search 50 adjacent genes that were significantly associated with NEK mutations in NSCLC patients and STRING11.0 database and Cytoscape to screen and visualize important module genes (Fig. 8A). Among them, CDK1, PLK1, CCNB1, and CCNB2 were significantly correlated with NEK mutations.

We used the DAVID database to analyze the NEKs and similar genes. Results of GO enrichment showed that molecular functions (MF), such as GO:0,003,777 (microtubule motor activity), biological processes (BP) such as GO:0,007,062 (sister chromatid cohesion), and cellular components(CC) such as GO:0,000,776 (kinetochore) were significantly regulated by NEK mutations in NSCLC (Fig. 8B). In addition, the pathway enrichment analysis showed that hsa04110 (cell cycle) and hsa04914 (progesterone-mediated oocyte maturation) were significantly related to the functions of NEKs in NSCLC (Fig. 8C).

## Discussion

The occurrence and development of lung cancer is a multi-stage process involving multiple genes and factors^31^. In addition to cancer genetics, abnormal epigenetic regulation at all stages plays an important role in its development^32^. It can be used as a reliable tool for early diagnosis and prognosis monitoring and as an effective therapeutic target for patients with lung cancer^33^. Although the roles of some members of the NEK family in lung cancer have been confirmed, investigating individual gene expression levels is not enough to evaluate the mechanism of lung cancer; therefore, the different roles of NEK family members in lung cancer still need to be clarified. In this study, we analyzed the differential expression, pathological parameters, mutations, and prognostic values of different NEK family members in NSCLC.

Results of this study showed that the NEK2/4/6/8 mRNA was overexpressed in LUAD patients, both in tumor stage and nodal metastasis status, and the highest expression level was mainly concentrated in phase 3/4; NEK2 mRNA was also overexpressed in LUSC patients, and the highest expression level was in phase 4; Therefore, we believe that the mRNA expression level of the above genes increases with the increase of tumor stage and nodal metastasis status. According to the clinical guidelines, the later the tumor stage and nodal metastasis status, the worse the prognosis. Therefore, it is speculated that NEK2/4/6/8 may become potential prognostic biomarkers for the survival of NSCLC patients. In addition, the expression levels of NEK1/3/4/5/6/7/8/9/10/11 proteins in NSCLC were higher than those in the normal group, which may be related to carcinogenesis, but NEK2 protein showed no expression in both normal group and NSCLC group, which may be due to the low expression levels of immune cells infiltrated in NSCLC. Furthermore, the high expression of NEK2/3 in NSCLC patients was significantly correlated with shorter overall survival time (OS), while the high expression of NEK1/4/6/7/8/9/10/11 mRNA in NSCLC patients was significantly correlated with good OS, which may be due to the fact that genes with high expression levels are more sensitive to treatment; hence, the prognosis is better. In addition, we observed high mutation rates of 56% and 53% in patients with LUAD and LUSC, respectively. NEK2/9 showed higher mutation in LUAD and LUSC patients, and mutation is a reason for the change of mRNA expression. Therefore, we speculated that NEK2/9 may interfere with mRNA expression by affecting DNA expression level and indirectly affect the prognosis of patients. Finally, CDK1, PLK1, CCNB1, and CCNB2 were significantly associated with NEK mutations. The GO enrichment analysis showed microtubule motor activity, ATP binding, and protein serine/threonine kinase activity in MF; sister chromatid cohesion, microtubule-based movement, and antigen processing and presentation in BP; and kinetochore, microtubule, and condensed chromosome kinetochore in CC. KEGG and RECTOME enrichment analysis showed that the main pathways were cell cycle- and progesterone-mediated oocyte maturation, which were significantly related to the functions of NEKs in NSCLC.

NEK2 is involved in mitosis and in the regulation of centrosome replication and spindle formation^34^. When the regulation is abnormal, chromosomal instability and aneuploidy occur, which are landmark changes in many tumors^35,36^, indicating that it plays an important role in the occurrence and development of tumors. At present, NEK2 is overexpressed in many cancers and is related to the poor prognosis of pancreatic ductal cancer^37^, prostate cancer^38^, and colon cancer^39^. In NSCLC patients with EGFR mutations, EGFR mutations can activate the ERK signal transduction pathway and then induce the high expression of NEK2, which can promote the rapid development of the cell cycle and the rapid proliferation of NSCLC cells, resulting in poor prognosis^40^. qRT-PCR experiments also showed that the expression level of NEK2 in LUAD tissues increased with the increase in TNM stage, and the high expression of NEK2 was significantly correlated with poor prognosis^41^. In this study, NEK2 was overexpressed in NSCLC, and it was significantly correlated with tumor stage, nodal metastasis status, and poor prognosis; this finding is consistent with the conclusions of the abovementioned study, indicating that NEK2 is more likely to be used as a prognostic marker of NSCLC, but further research is needed.

In the process of cancer development, NEK4 can repair defects by inhibiting DNA, which makes cancer cells sensitive to apoptosis^42^. In vitro experiments have shown that knocking down NEK4 can reduce the expression of transcription factors Zeb1 and Smads, which play key roles in the process of epithelial-mesenchymal transition (EMT). Meanwhile, EMT and its intermediate state play important roles in tumor invasion and metastasis; hence, NEK4 knockout can reduce the metastasis rate of lung cancer cells^43^. In addition, the lack of NEK4 can reduce the expression of survivin^44^; survivin is an anti-apoptotic protein highly expressed in most cancers, which can increase the tumor recurrence rate and reduce the survival rate of patients^38,45^.

NEK6 can regulate many cell processes, such as cell cycle, apoptosis, and senescence^46^, and accelerate the entry of tumor cells in the S phase^47^. The expression level and activity of NEK6 in mitosis of many types of malignant tumors can be increased^48,49^, which can inhibit the premature senescence of cancer cells induced by p53 and promote the occurrence and development of tumors^47^. Previous studies have shown that NEK6 is significantly upregulated in breast cancer and may play a role in the proliferation of breast cancer cells. In addition, the expression of NEK6 was positively correlated with histological grade, tumor size, and TNM stage of breast cancer; therefore, we believe that NEK6 may be an important index for predicting the prognosis of patients with breast cancer^46^. Some studies have also shown that NEK6 is overexpressed in advanced gastric cancer and in 70% of liver cancer patients^50,51^. Animal experiments have also shown that low expression levels of NEK6 can lead to tumor cell reduction^52^.

NEK8 plays a role in the cell cycle G2-M phase^53^ and the DNA damage response signaling pathway^53,54^. It can play a potential role in tumorigenesis and DNA damage response through the MYC proto-oncogene^55^ and serine/threonine kinase signaling pathway^54^. NEK8 may be the target gene of hypoxia-inducible factor (HIF), whereas von Hippel-Lindau tumor suppressor protein may downregulate the expression of NEK8 through the HIF^56^. In addition, some studies have found that NEK8 is overexpressed in breast cancer^57^.

In this study, NEK4/6/8 was highly expressed in LUAD, which was closely related to tumor stage and nodal metastasis status. However, their high expression levels were associated with good prognosis, probably because the high expression level made these genes more sensitive to treatment; hence, the prognosis was better.

NEK1/10/11 is related to DNA damage response^34^. Among them, the expression level and activity of NEK1 are increased during mitosis^58,59^. When it is mutated or silenced, spindle defects, abnormal chromosome segregation, mitotic block, and apoptosis occur; therefore, NEK1 plays an important role in the development of cancer^34,58–60^. NEK1 can participate in the regulation of DNA damage repair in HeLa cervical cancer cells^61^, and its overexpression can significantly reduce disease-free survival^62^; it can also be overexpressed in human glioma tissues, which is related to tumor grade and nodal metastasis status^63^. A decrease in NEK10 expression can lead to increased cell proliferation and DNA replication^64^, which is related to poor prognosis and higher tumor grade of breast cancer^65^. As part of the physiological barrier induced by DNA damage, NEK11 was overexpressed in precancerous lesions of 35% of colorectal adenomas and colon cancer, and its high expression level is associated with low tumor grade and weak invasive ability^66,67^. NEK11 is a potential tumor suppressor gene^68^ and is significantly downregulated in ovarian cancer mediated by methylation or mutation^69,70^. In this study, however, the low expression of NEK1 in NSCLC was significantly correlated with poor prognosis, which was inconsistent with the above results, which may be due to the decrease in the overall average expression level of immune cells with low expression levels in NSCLC. The results of NEK10/11 are consistent with those of the abovementioned study; the higher the expression levels, the better the prognosis.

Some studies have found that NEK3 can regulate cell migration, proliferation, viability, and neuronal development^69–72^ and may play an important role in the occurrence and development of cancer^73^. The expression level of NEK3 is related to the invasiveness, TNM stage, and tumor size of thyroid carcinoma^14^. In addition, NEK3 is overexpressed in gastric cancer, which is closely related to pT stage, TNM stage, and nodal metastasis status, and can significantly reduce OS and disease-free survival in patients with gastric cancer^74^. NEK3 is also highly expressed in invasive breast cancer, which can promote the migration and invasion of breast cancer, and inhibition of its activity can lead to loss of invasive phenotype^75^. However, in this study, the low expression of NEK3 in NSCLC may be due to the loss of expression during the transformation of lung malignant tumors^16^, and its high expression level is significantly correlated with shorter OS. Some studies have suggested that NEK5 is also related to the development of cancer and can interact with caspase-3^76^. Caspase-3 is a protease involved in cell apoptosis and differentiation; therefore, NEK5 is also involved in cell death^13,76^. The upregulation of NEK5 is significantly related to the progression and poor prognosis of breast cancer. When NEK5 is silenced, it can significantly prevent the proliferation of breast cancer cells in vivo and in vitro, thus inhibiting their migration and invasion^15^. In this study, we found that the expression level of NEK5 in NSCLC is relatively low; moreover, no study has investigated its prognostic value. Due to the lack of research on NEK5, its importance in the occurrence and development of cancer cannot be confirmed.

NEK9/7/6 can be activated during mitosis and jointly regulate spindle formation^77^, in which activated NEK9/7 can change the microtubule dependence of NSCLC cell morphology and promote its migration^78^. NEK9 is thought to play a role upstream of NEK7/6^79^, and its main function is to activate NEK7/6 through various mechanisms such as activated cyclic phosphorylation, autophosphorylation induced by dimerization, and catalytic site recombination induced by allosteric binding^79–82^. For example, NEK9 can directly interact with NEK7 through a short sequence (residues 810–828) located in its C-terminal region between the RCC1-like domain (residues 347–726)^78^ and the RCC1-like domain C-terminal coiled helix motif (residues 891–-939)^83^ and may interact with NEK6^81^. The carcinogenic driver EML4-ALK^84^ is detected in 5% of NSCLC patients, and the high expression of NEK9 can lead to poor progression-free survival^78^. NEK7 can be highly expressed in gallbladder carcinoma, which is closely related to tumor differentiation, Nevin stage, and metastasis, and can significantly shorten the OS^85^. At present, a few studies have investigated the expression levels, prognostic values, and interactions of NEK9/7 in tumor cells; in this study, after analyzing the expression levels of NEK9/7 in NSCLC and normal tissues, it was found that NEK9/7 is highly expressed in normal tissues and is closely related to a good prognosis.

In summary, our results show that the mutation rates of NEK family genes in NSCLC are relatively high. In LUAD patients, the mutation rate was 56%, in which NEK2/4/6/8 genes were overexpressed. In LUSC patients, the mutation rate was 53%, in which NEK2 was overexpressed; all of them were significantly related to tumor stage and nodal metastasis status. In addition, the high expression of NEK2/3mRNA was significantly associated with poor prognosis in patients with NSCLC, while the high expression of NEK1/4/6/7/8/9/10/11 mRNA was associated with good prognosis. These results suggest that NEK2/4/6/8 may be a prognostic biomarker for the survival of patients with NSCLC. However, since this study only evaluated the relationship between NEK mRNA expression level and survival rate and did not consider the limitations of other confounding factors, the results of survival analysis are still uncertain, and further studies are needed to verify these results.

## Data Availability

All data generated or analyzed during this study are included in this published article.
